# Structure, gene regulation and environmental response of flagella in *Vibrio*

**DOI:** 10.3389/fmicb.2013.00410

**Published:** 2013-12-25

**Authors:** Shiwei Zhu, Seiji Kojima, Michio Homma

**Affiliations:** Division of Biological Science, Graduate School of Science, Nagoya UniversityNagoya, Japan

**Keywords:** bacterial flagellum, ion-driven motor, motility, chemotaxis, pathogenicity

## Abstract

*Vibrio* species are Gram-negative, rod-shaped bacteria that live in aqueous environments. Several species, such as *V. harveyi*, *V. alginotyticus*, and *V. splendidus*, are associated with diseases in fish or shellfish. In addition, a few species, such as *V. cholerae* and *V. parahaemolyticus*, are risky for humans due to infections from eating raw shellfish infected with these bacteria or from exposure of wounds to the marine environment. Bacterial flagella are not essential to live in a culture medium. However, most *Vibrio* species are motile and have rotating flagella which allow them to move into favorable environments or to escape from unfavorable environments. This review summarizes recent studies about the flagellar structure, function, and regulation of *Vibrio* species, especially focused on the Na^+^-driven polar flagella that are principally responsible for motility and sensing the surrounding environment, and discusses the relationship between flagella and pathogenicity.

## Introduction

**Figure 1:**
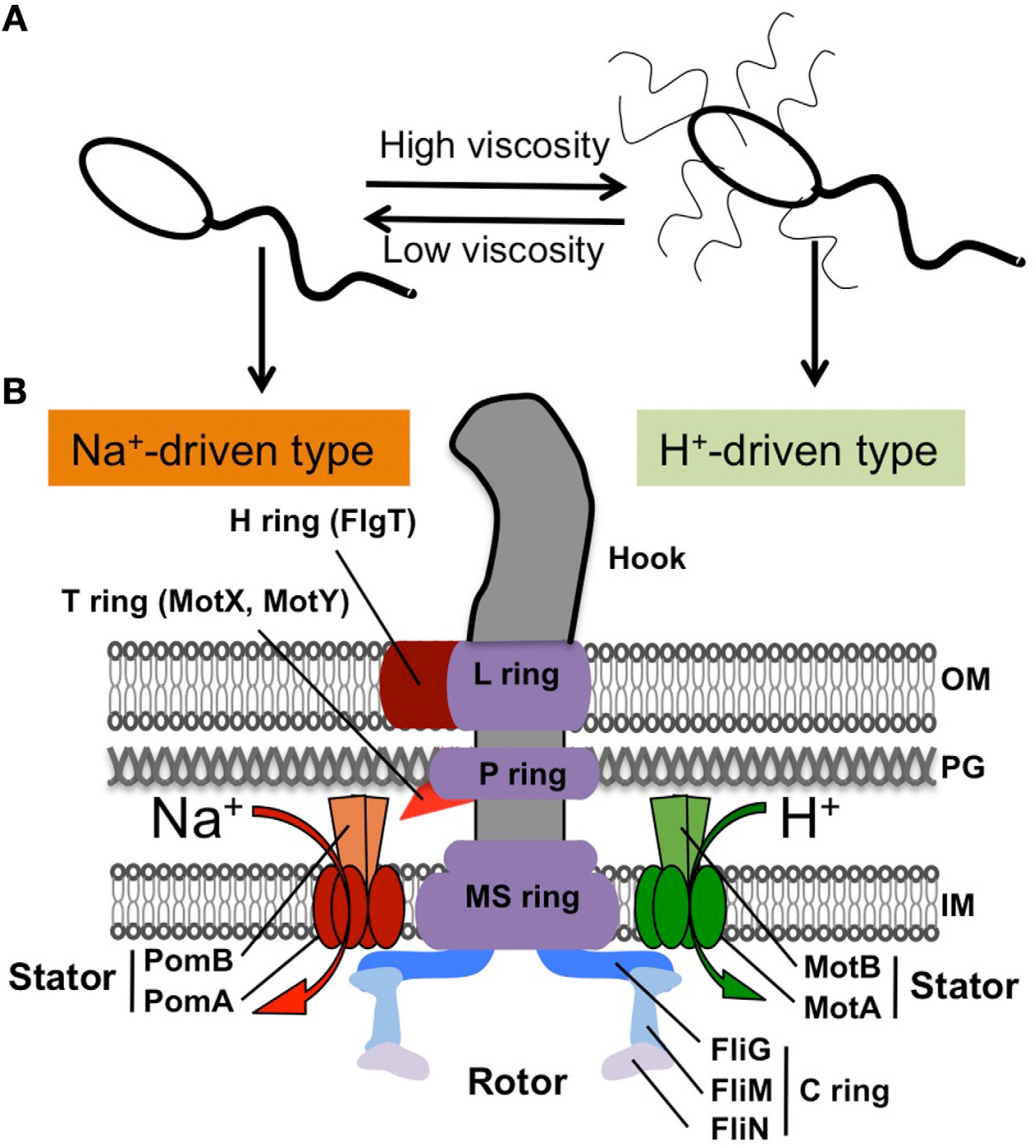
**Flagellar structure of *Vibrio*. (A)** Polar flagellum and lateral flagella in some *Vibrio* species (not including *Vibrio cholerae*). **(B)** Schematic diagram of the hook basal body for a polar flagellum shown in the left half (based on EM images, (Terashima et al., 2006, 2013)) and the lateral flagellar structure shown in the right half, according to the peritrichous flagellum from *Salmonella* (MacNab, 2003). The characteristics of the polar flagellum are the H ring and the T ring, shown in red.

*Vibrio* species are Gram-negative, rod-shaped bacteria that live in all types of aqueous environments, including marine, freshwater, and estuary (Blake et al., 1980; Joseph et al., 1982; Johnson et al., 2012). All *Vibrio* species can move using flagella, which are cell surface organelles that can propel them. Unlike eukaryotic flagella, each bacterial flagellum is driven by a rotary motor embedded in the cell envelope, and the flagellar rotation is harnessed by the ion-motive force across the cell membrane (Berg, 2003; Terashima et al., 2008). The basic structure and function of the flagellum produced by *Vibrio* species are the same as those in other species (Figure 1) (Aizawa, 1996; Chen et al., 2011). Each flagellum consists of a filament acting as a helical propeller, a hook functioning as a universal joint and a basal body working as a rotary motor (Figure 1) (Sowa and Berry, 2008; Li et al., 2011). More than 50 gene products are involved in flagellar synthesis (MacNab, 2003). Since flagella are relatively large motility organelles for the cell, the formation of the flagella and the expression of their components are tightly regulated. Their assembly process has been described in many reviews (Chilcott and Hughes, 2000; Kim and McCarter, 2000; Aldridge, 2002; Chevance and Hughes, 2008).

All *Vibrio* species have single or multiple flagella at the cell pole (called “polar flagellum”) and can swim freely in a liquid environment. With respect to the flagellum, *V. cholerae* has a single polar flagellum (monotrichous). However, some species, such as *V. parahaemolyticus* and *V. alginolyticus*, produce enormous numbers of flagella at lateral or peritrichous positions (called “lateral flagella”) in addition to the single polar flagellum when they are exposed to viscous environments (Figure 1A) (McCarter et al., 1988; Kawagishi et al., 1996). The polar and lateral flagella are structurally and functionally distinct from each other (McCarter, 2004). Each polar flagellum is covered with a sheath that is contiguous with the outer membrane, so that it is thick and can be observed using dark-field microscopy with a mercury lamp. Lateral flagella are not covered with a sheath, so they are difficult to observe using light microscopy. Although both types of flagella are driven by rotary motors embedded at their bases, the power source is different. Polar flagella are driven by the Na^+^-motive force, whereas lateral flagella are driven by the H^+^-motive force, as seen in the lateral flagella of *Escherichia coli* or *Salmonella enterica* (Figure 1) (Atsumi et al., 1992; Asai et al., 2000; Blair, 2003).

In terms of the pathogenicity of *Vibrio*, some species, such as *V. cholerae*, *V. vulnificus*, and *V. parahaemolyticus*, have been described extensively as human pathogens (Daniels and Shafaie, 2000; Yildiz and Visick, 2009), and *Vibrios* are also pathogenic to fishes or the other animals. In this review, we focus on flagellar function and assembly and on the relationship between flagellar motility and pathogenicity.

## Flagellar basal body structure and motor

The overall structure of the flagellar base is shown schematically in Figure 1B, based on electron microsopic images of the purified hook-basal body from a peritrichous flagellum of *Salmonella enterica* (right side) and from a polar flagellum of *Vibrio alginolyticus* (left side) (Francis et al., 1994; Thomas et al., 2006; Terashima et al., 2008, 2013). Both kinds of basal bodies share common features even though they originate from different species of Gram-negative bacteria: the hook and basal body with several rings embedded in the cell envelope. The flagellar basal body functions as a rotary motor, and consists of two parts: the rotary part (rotor) and the stationary part (stator). The stator complex is composed of two proteins, MotA/MotB (LafT/LafU), for the H^+^-driven motor of lateral flagella from *E. coli*, *Salmonella* and *Vibrio*, and PomA/PomB, for the Na^+^-driven polar flagellar motor of *Vibrio* (Figure 1). The ion flux through the stator couples to the rotor-stator interaction that generates torque. The rotor contains several rings: from the cytoplasmic face, there is the C ring (also called the “switch complex”) composed of FliG, FliM, and FliN, and the MS ring embedded in the cytoplasmic membrane (made of at most 26 copies of FliF). These rings are connected by a rod whose tip connects to the hook. The basal body contains two other rings, the P ring (FlgI), which is associated with the peptidoglycan layer, and the L ring (FlgH), which is located in the outer membrane (Aizawa, 1996; Terashima et al., 2008). Thus, the LP ring does not rotate but functions as a bushing for the central rod.

**Figure 2:**
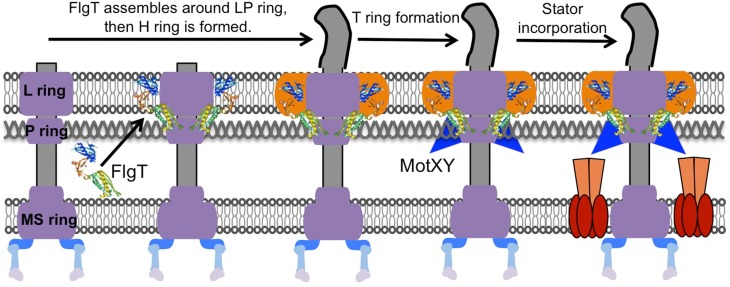
**Model for the basal body ring formation of the Na^+^-driven polar flagellum**. The H ring is constructed dependent on FlgT proteins that assemble around the previously formed L ring and P ring. Next, MotX and MotY assemble around the basal body to stably form the T ring with the help of the middle domain of FlgT. As a result, the stator is incorporated properly and works in the presence of the T ring. The model is drawn based on a previous report (Terashima et al., 2013).

Although high resolution ultrastructural images have been reported for the basal body, intact images of the entire flagellar motor have remained ambiguous until recently due to the complexity of the stator units, which dynamically assemble around the rotor (Leake et al., 2006). However, the stator always dissociates from the detergent-solubilized flagellar basal body and no one has been able to isolate the basal body intact with the stator. In 2006, Murphy and co-workers (Murphy et al., 2006) first showed the structure of the complete flagellar motor *in situ* using the whole cell electron cryotomography method from the spirochete *Treponema primitia.* Their images captured electron densities corresponding to the stator units surrounding around the rotor. The resolution was still not sufficient to identify the detailed structure of the stator, but those images revealed the relative locations of the C-ring and the stator units for the first time. The structure of the polar flagellum of *Vibrio* contains two additional ring structures, named the T ring, which surrounds the periplasmic side of the P ring (Terashima et al., 2006), and the H ring, which is located at the outer rim of the L ring. The T ring is made of two proteins, MotX and MotY, that are essential components for the polar flagellar motor of *Vibrio*, and it is probably involved in stabilizing the stator surrounding the rotor (McCarter, 1994a,b; Okabe et al., 2001; Terashima et al., 2006). The crystal structure of MotY from *V. alginolyticus* in conjunction with biochemical analysis revealed that the C-terminal domain of MotY stabilizes the association with the peptidoglycan layer and that the N-terminal domain of MotY is involved in the association with the basal body (Kojima et al., 2008). The H ring is located at the outer rim of the LP ring, so that we initially thought that the LP ring was somehow bigger in this basal body (Figure 2). The loss of *flgT* function results in an almost non-motile phenotype and there is no formation of the T ring in addition to the lack of an outer rim structure of the LP ring. Therefore, FlgT is likely to be a component of the H ring, and is also important for assembly of the T ring. Our crystal structure and functional analyses of FlgT support this idea (Terashima et al., 2013). Perhaps these two unique extra ring structures in the polar flagellum are required to fix the stator units around the rotor (Figure 2). The *Vibrio* polar flagella can rotate extraordinarily up to 1700 Hz (Magariyama et al., 1994), while the rotational speed of the H^+^-driven flagellar motor is just around 300 Hz (Chen and Berg, 2000).

It is noteworthy that in the *E. coli* H^+^-driven motor, unexpectedly dynamic properties of the stator units were observed by fluorescent photobleaching experiments: the stator units are not really static components of the motor, but can dynamically associate with and dissociate from the rotor (Leake et al., 2006). Such a dynamic characteristic can be observed also for the PomA/PomB stators of the *Vibrio* polar flagellum. Their assembly around the rotor is dependent on the external Na^+^ concentration (Fukuoka et al., 2009). Removal of Na^+^ from the medium caused the dissociation of the stators from the rotor, and the subsequent addition of Na^+^ to the medium restored the stator incorporation to the motor. The physiological meaning of the dynamic property of the stator is not known, but the rapid and stable rotation of the motor may be compensated by the turnover of the units to components pooled on the membrane.

## Flagellar gene regulation and assembly

About 50 gene products are involved in the construction of a functional flagellum. Since the flagellum is such a big organelle and its production and assembly requires a large commitment of resources, especially for filament formation, bacteria have developed a precise regulation system that controls flagellar construction (MacNab, 2003). The control mechanism has been well-characterized in the system of *Salmonella* flagella (Chilcott and Hughes, 2000). In brief, the flagellum assembles from the inner structure base to the outer ones, beginning with basal body construction followed by hook assembly, and finally by filament formation. Therefore, any defects in gene products that disrupt the basal body or the hook formation inhibit the filament assembly. The hook assembly step is the critical check point: if the hook subunits are polymerized to the proper length (about 55 nm), then genes required for filament formation are induced. This assembly-coupled flagellar gene regulation is achieved by a cascade of flagellar gene operons (called the flagellar regulon). In *Salmonella*, there are three classes of operons: early, middle, and late (Kutsukake et al., 1990). The master regulator for the flagellar regulon (FlhDC) belongs to the early operon that induces the expression of the middle operon. The middle operon contains genes encoding for basal body and hook proteins. It also contains regulator σ^28^ (FliA) that controls the expression of genes belonging to the late operon, which encodes filament and motor proteins (Ohnishi et al., 1990). After completion of the hook assembly, σ^28^ is released from anti-sigma factor FlgM binding, and so is able to induce the expression of genes in the late operon (Ohnishi et al., 1992).

**Figure 3:**
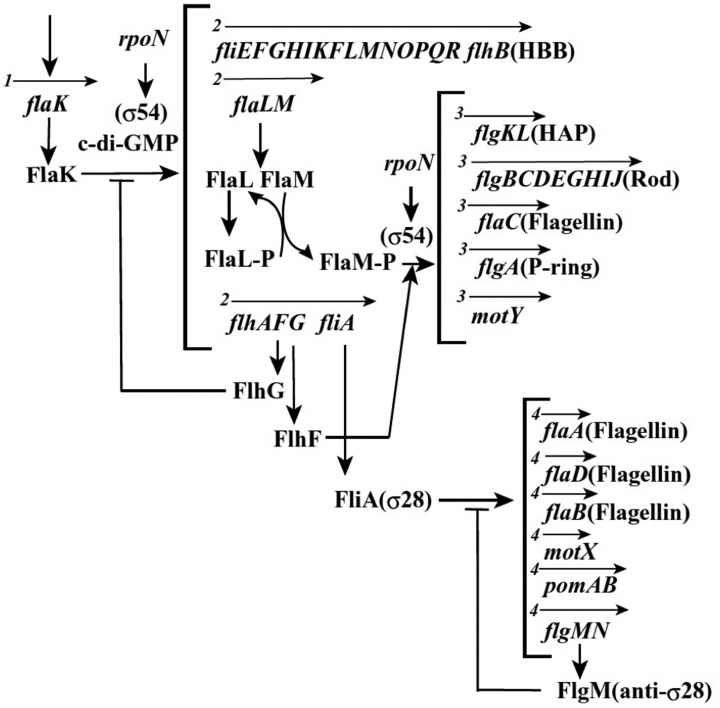
**Plausible hierarchy of polar flagellum gene expression in *Vibrio*, based on previous reports (McCarter, 2001; Correa et al., 2005; Kojima et al., 2011)**. The arrows indicate the transcription unit and the number at the beginning of arrow indicates the class of transcriptional hierarchy.

In *Vibrio*, the morphogenetic pathway for the polar and lateral flagella is quite likely to be the same as the one identified for *Salmonella*. However, the gene regulation for polar flagellar synthesis is more complex (Figure 3). Detailed analyses have been carried out for *V. cholerae* and *V. parahaemolyticus* and have found that the flagellar regulon is composed of a combination of both a RpoN (σ^54^) and a FlrA/FlaK (master regulator) dependent transcriptional hierarchy, organized into four classes (Klose and Mekalanos, 1998; Klose et al., 1998). Distinct from the *Salmonella* cascade, the middle operons are divided into two classes (class 2 and 3), and two sigma factors are involved in the regulation (Kim and McCarter, 2000). σ^54^ controls class 2 and 3 operons, which encode basal body and hook components, and σ^28^ (FliA) controls class 4 operons, which encode filament, chemotaxis, and motor components (Stewart and McCarter, 2003). Since *V. alginolyticus* is so closely related to *V. parahaemolyticus*, it is likely to have a similar mechanism that regulates the polar flagellar synthesis, and the putative organization of its polar flagellar regulon is shown in Figure 3 (McCarter, 2001; Correa et al., 2005; Kojima et al., 2011). It is noteworthy that *Vibrio* species have two chromosomes: the larger one contains all genes involved in the polar flagellar systems, whereas the smaller one contains all genes for the lateral flagellar systems (Makino et al., 2003). Recent work that the flagellar regulatory hierarchy facilitates the correct spatiotemporal expression patterns for optimal *V. cholerae* colonization and disease progression, is consistent with the idea that motility and the expression of specific virulence factors are inversely regulated (Syed et al., 2009).

**Figure 4:**
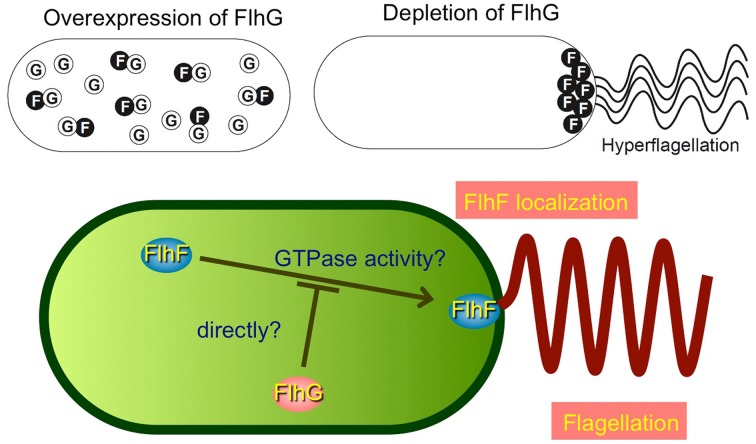
**Model for regulation of the number of polar flagella in *Vibrio***. The function of FlhF is probably inhibited by an interaction with FlhG, a negative regulator of FlhF, and thereby the number of polar flagellum is limited to one. The schemes are drawn based on a previous report (Kusumoto et al., 2008).

Another interesting topic regarding polar flagellar assembly is the regulation of their number and placement: *V. alginolyticus* cells have only a single polar flagellum at the cell pole. Genetic analyses identified two proteins, FlhF and FlhG, which are involved in this regulation (Kusumoto et al., 2008). FlhF is a GTPase and signal-recognition particle (SRP) homolog that positively regulates the number of polar flagella, and also determines the polar positioning (Salvetti et al., 2007; Balaban et al., 2009). FlhG is a MinD homolog and a putative ATPase that negatively regulates the flagellar number (Kusumoto et al., 2006). Biochemical and protein localization studies using GFP fusion derivatives revealed that FlhG binding to FlhF prevents the polar localization of FlhF, so that the proper number of FlhF is localized at the pole to initiate a single polar flagellum (Figure 4) (Correa et al., 2005; Kusumoto et al., 2008). From the *flhF flhG* double mutant, which mostly lacks flagella, a suppressor strain was isolated that forms peritrichous flagella in the majority of cells (Kojima et al., 2011). A mutation was recently found in a *dnaJ* family gene, named *sflA* (suppressor of Δ*flhFG*), which changed the monotrichous flagellar *Vibrio* cells into peritrichous flagellated cells (Kitaoka et al., 2013). SflA protein is proposed to prevent the initiation of flagellar construction.

## Sensing the environmental conditions

**Figure 5:**
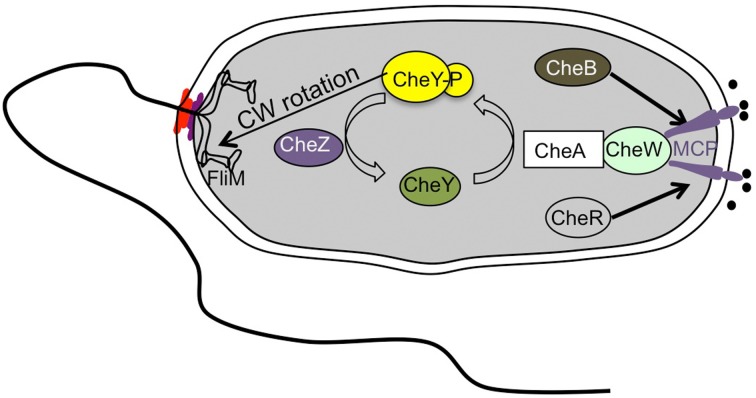
**Simplified scheme of the chemotaxis signal transduction cascade in *Vibrio* species**. The scheme is drawn based on a previous report (Boin et al., 2004).

As seen in other bacteria like *E. coli*, *Vibrio* species also show chemotaxis, moving toward favorable conditions and avoiding unfavorable environments (Figure 5) (Szurmant and Ordal, 2004). The chemotaxis-related genes have been well-characterized for *V. cholerae.* Whole genome sequence analysis predicted three *che* gene clusters: cluster I and II on chromosome I and cluster III on chromosome II (Heidelberg et al., 2000). Although many homologs of various chemotaxis components found in *E. coli* have been identified, only one of three chemotaxis operons, cluster II, is required for chemotaxis in *V. cholerae* (Gosink et al., 2002). Current understanding of chemotactic behavior using a polar flagellar system is similar to that of *E. coli* (Figure 5). Once an attractant chemical binds to the periplasmic region of the methyl-accepting chemotaxis proteins (MCPs), a conformational change is generated that triggers autophosphorylation of the cytoplasmic kinase CheA, which is assumed to be associated with MCPs via adaptor protein CheW. Consequently, CheA donates the phosphate group to a response regulator CheY that triggers a rotational switch from counter-clockwise to clockwise by binding to FliM, a component of the switch complex. Meanwhile, some specific residues in the cytoplasmic domain of the MCP will be in a dynamic state of methylation with the help of CheR, a constitutively active methyltransferase, and of CheB, a methylesterase activated by phosphorylation. Methylated MCPs cease the signal output, so that cells are back to the original state (Boin et al., 2004).

The number of chemotaxis genes in *V. cholerae* is much greater than in *E. coli*, but only some of them are functional under experimental conditions (Banerjee et al., 2002). In *V. cholerae*, only deletion of *cheA-2* in cluster II on chromosome I impairs chemotaxis and the *che* genes in this cluster are the most homologous to the *che* genes in other *Vibrio* species, suggesting that *che* cluster II is involved in chemotaxis. Moreover, only the Che-Y3 protein among the 5 *cheY* paralogs showed a relationship to flagellar rotation (Boin et al., 2004; Hyakutake et al., 2005). Furthermore, there are 45 proteins predicted to be potential chemoreceptors. However, only some chemoreceptors such as Mlp24 (*m*ethyl-accepting chemotaxis *l*ike *p*rotein, MCP homologs) recently reported were implicated in pathogenicity (Nishiyama et al., 2012). Thus, multiple chemotaxis-related genes and a large number of predicted MCP-like proteins imply a distinct chemotaxis mechanism that allows *V. cholerae* to adapt to various environmental conditions. However, Hyakutake et al. suggested that *che* clusters I and III are perhaps involved in controlling function(s) other than chemotaxis (Hyakutake et al., 2005). With regard to chemotaxis and pathogenicity, a non-chemotactic strain with counter-clockwise-biased flagellar rotation displays increased colonization and infectivity whereas reduced competition of a clockwise-biased strain was found (Butler and Camilli, 2004, 2005). A global transcription profile approach from stool samples showed that expression of many chemotaxis genes is at a low level, but some of them (*cheA-2*, *cheY-3*, *cheW-1*, and *cheR-2*) are not altered (Merrell et al., 2002). CheA-1 is highly expressed during human infection (Hang et al., 2003). Furthermore, it has been reported that human-shed *V. cholerae* have a 10-fold lower oral infectious, and have a transiently reduced chemotactic state in which the protein level of CheW was reduced (Butler et al., 2006). This shows that a pathogen alters its chemotactic state in response to human infection.

In addition to the chemotaxis, which mediates the direction of flagellar rotation, the polar flagellum seems to be a mechanosensor. As described above, *V. alginolyticus* and *V. parahaemolyticus* induce numerous lateral flagella in response to increases in external viscosity. The details of this sensing mechanism are still not understood. Besides an increase in viscosity, lateral flagellar expression is also induced by the addition of an anti-polar flagellum antiserum (McCarter et al., 1988). Since polar flagellar rotation is restricted in these two conditions, the polar flagellum also functions as a mechanosensor to induce lateral flagellar systems. Moreover, it was reported that inhibition of polar flagellar rotation by the specific inhibitor phenamil could induce lateral flagellar expression in media devoid of viscous agents, indicating the possibility that *Vibrio* cells can sense a decrease in the rotation rate of (or sodium influx through) the polar flagellar motor as a trigger for lateral gene expression (Kawagishi et al., 1996). Recently, it was reported that the mechanosensing mechanism by flagella is due to the dynamic assembly of the stator in *E. coli* (Lele et al., 2013; Tipping et al., 2013). Tipping and colleagues showed that the number of the stators bound to the flagellar motor is dependent on the external mechanical load, with more stators at higher viscosity and fewer stators at lower loads (Tipping et al., 2013). The flagellar motor of bacteria senses external load and regulates the strength of stator binding to the rest of the motor. Through this process the flagella can sense the external viscosity, suggesting that they function as a mechanosenseor as well as a locomotion component, and impact cellular functions (Lele et al., 2013). In this regard, it is suggested that the polar flagellum in *Vibrio* perhaps applies the same mechanosensing mechanism to sense and respond to its environment.

## Pathogenesis and motility

Of particular interest to the pathogenesis of *Vibrio* is to determine the relationships between virulence factors, motility, and biofilm formation. *V. cholerae*, *V. parahaemolyticus*, and *V. vulnificus*, in addition to fish pathogens like *V. alginolyticus* have been established as important pathogens (DePaola et al., 2003; Xie et al., 2005; Jones and Oliver, 2009; Rasmussen et al., 2011; Kim et al., 2013; Ren et al., 2013; Sreelatha et al., 2013). In *V. cholerae*, the major virulence factors are cholera toxin (CT) and toxin co-regulated pilus (TCP), which are mediated in response to environmental stimuli through a hierarchical regulatory cascade called the ToxR regulon (Childers and Klose, 2007). ToxR and TcpP, two membrane-localized transcription factors, activate the expression of ToxT, another important transcription factor in the cytoplasm, which results in the activation of CT and TCP (DiRita et al., 1991; Krukonis and DiRita, 2003). CT can cause severe diarrhea in humans, and is encoded in the CTXΦ prophage (Hassan et al., 2010). A large *Vibrio* pathogenicity island (VPI), encoded in the VPIΦ prophage (Karaolis et al., 1998), is required for the TCP gene cluster, functioning both as an essential colonization factor and as a CTXΦ receptor (Taylor et al., 1987; Herrington et al., 1988; Lowden et al., 2010). However, recent research has shown that the ToxR cascade is also involved in repressing the production of virulence factors, suggesting that the ToxR regulon in *V. cholerae* may play a broader role in pathogenesis (Bina and Bina, 2010; Bina et al., 2013).

With regard to the pathogenesis of *V. parahaemolyticus*, a wide variety of virulence factors, including adhesins, thermostable direct hemolysin (TDH), TDH related hemolysin and Type 3 Secretion Systems, have been well-described by Zhang and Orth (2013). Interestingly, although hemolysin is thought to be a major virulence factor (Makino et al., 2003; Zhang and Austin, 2005), a deletion of the gene encoding both hemolysins does not drastically affect its cytotoxic effects on cultured cells (Zhang and Orth, 2013), suggesting that other virulence factors play important roles in the pathogenesis of* V. parahaemolyticus*. With regard to the pathogenesis of *V. vulnificus*, a recent review described that it possesses a variety of virulence factors, including capsular polysaccharide, acid neutralization, iron acquisition, acid neutralization and expression of proteins involving in motility, attachment and adhesion (Jones and Oliver, 2009).

Several studies have probed the relationship between the motility and the pathogenicity of *V. cholerae in vivo* (Gardel and Mekalanos, 1996; Krukonis and DiRita, 2003; Silva et al., 2006; Syed et al., 2009). It was initially thought that the expression of motility genes may repress the virulence genes (Gardel and Mekalanos, 1996). Mutational analyses have shown that motility is a virulence determinant, since non-motile mutants do not adhere to isolated rabbit brush borders and non-motile strains seem to have attenuated virulence for humans (Richardson, 1991; Silva et al., 2006). However, non-motile mutants have no significant defect in the ability to colonize suckling mice, implying that motility does not affect colonization dependent on TCP (Silva et al., 2006). The expression of motility genes is likely to repress virulence genes and vice-versa (Gardel and Mekalanos, 1996). Subsequently, Häse and coworkers showed that reducing the motility of *V. cholerae* by increasing the medium viscosity or disrupting the Na^+^-motive force results in an increase in the expression of ToxT (Häse and Mekalanos, 1999). It is noteworthy that the flagellar stator as a mechanosensor responds to assembly around the flagellar rotor in the case of high viscosity and consequently conducts massive Na^+^ ions influx. Meanwhile, a large number of Na^+^ ions are inside of cell in the case of disrupting the Na^+^-motive force. Thus, we can speculate that the influx of Na^+^ ions through the flagellar stator regulates the expression of ToxT.

**Figure 6:**
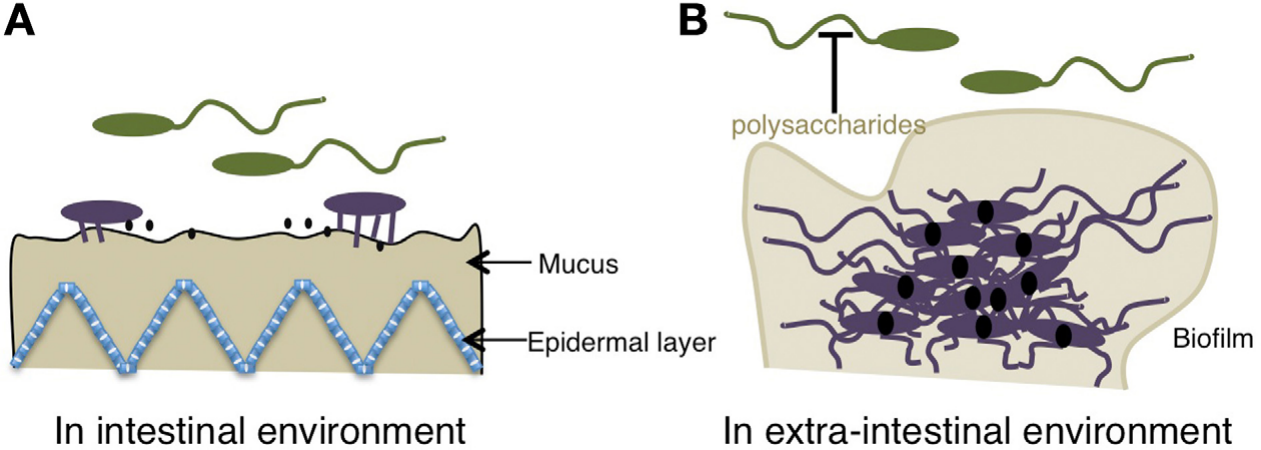
**Relationship between the motility of the polar flagellum of *Vibrio* and pathogenicity. (A)** In an intestinal environment, because of the presence of bile, the gene expression of virulence is “OFF.” When the polar flagellum senses the high viscosity of the mucus gel, the motility is blocked, lateral flagella or pili are induced, and pathogenic gene expression, including toxins (black solid dots) is kept “ON” presumably because of the reduced bile concentration. **(B)** In an extra-intestinal environment, c-di-GMP (solid black dots) facilitates *Vibrio* from the motile-to-sessile transition by decreasing migration due to the binding of the YcgR homolog to the flagellar motor, which causes a reduction of motility and induces the biofilm formation and the suppression on the motility by releasing a lots of polysaccharides. The schemes are drawn based on previous reports (Krasteva et al., 2010; Boyd and O'Toole, 2012).

With regard to the motility and the pathogenicity, Silva et al. found a remarkable increase in CT and TCP major subunit in a non-motile strain (*motY*), but a decrease in CT production in both wild-type and mutant strains when flagellar motility was inhibited (Silva et al., 2006). Recent work revealed a clear contribution of the flagellar regulatory hierarchy to the virulence of *V. cholerae* (Syed et al., 2009). Virulence factors such as toxin and hemolysin, which are up-regulated in flagellar regulatory mutants, were confirmed by quantitative reverse transcription PCR. The flagellar regulatory system positively mediates the transcription of diguanylate cyclase, named CdgD which results in the transcription of a hemagglutinin that enhances intestinal colonization (Syed et al., 2009). Thus, this whole-genome expression analysis supports the concept that motility and virulence gene expression are inversely regulated. Meanwhile, it has been found that TCP is expressed before the generation of CT during infection (Lee et al., 1999). These findings specify another transitional phase from motility to pathogenicity: colonization prior to release of CT (Figure 6A).

A similar relationship between motility and pathogenicity can be found between motility and biofilm formation in the extra-intestinal environment (Figure 6B). Once *Vibrio* cells are colonized, they form a biofilm and are encapsulated and thereby protected (Costerton et al., 1999). Biofilm formation is an important lifestyle of pathogenic *Vibrio* and renders bacteria resistant to environmental stresses, such as antimicrobial compounds or drugs (Yildiz and Visick, 2009; Lasarre and Federle, 2013). Biofilm formation in *V. cholerae* is controlled by quorum sensing (QS) through the modulation of cyclic di-guanosine monophosphate (c-di-GMP) that is used by bacterial pathogens to regulate the expression of genes involved in defense and invasion (Hammer and Bassler, 2003; Zhu and Mekalanos, 2003; Waters et al., 2008). A role of c-di-GMP in biofilm and motility is becoming clear. A *flaA motX* double mutant formed smooth colonies, indicating that flagella are involved in the initial stages of biofilm formation (Lauriano et al., 2004). For biofilm formation in *V. cholerae*, VpsT, a transcriptional regulator, has been reported to control *Vibrio* polysaccharide gene expression and to inversely regulate biofilm formation and motility, via c-di-GMP (Krasteva et al., 2010). Polysaccharide is a very important factor regarding the pathogenicity of *Vibrio* in the case of immune evasion and biofilm formation (Figure 6B) (Waldor et al., 1994; Naka et al., 2011; Johnson et al., 2012).

Regarding motility, several groups have shown that in *E. coli* and* Salmonella*, c-di-GMP directly bound to YcgR interacts with components of the flagellar motor to disrupt flagellar rotation, thereby leading to decreased motility (Boehm et al., 2010; Fang and Gomelsky, 2010; Paul et al., 2010). Since homologs of the YcgR protein that contains a binding domain of c-di-GMP extensively exist in *Vibrio*, the same impacting mechanism for the transition from the motile to the sessile phenotype by regulating c-di-GMP also exists in *Vibrio* (Pratt et al., 2007). Interestingly, on one hand, flagellar motility is involved in biofilm formation in the initial stage, but on the other hand, it is repressed when the biofilm is formed. This perhaps implies much broader functions for flagella in addition to motility and mechanosensing. Further, QS also influences motility and polar flagellar biogenesis via a master regulator for QS, SmcR that down-regulates *flhF* expression at the transcriptional level (Kim et al., 2012). Following the loss of motility and the formation of bacterial community, the responses induced by QS signals are usually involved as virulence determinants, such as toxins, proteases, polysaccharides and other relative fitness factors (Rutherford and Bassler, 2012).

## Summary

Overall, this Review has focused on the structure, gene regulation and sensing of the polar flagellum in *Vibrio* spp., and emphasizes the inverse relationship between motility and pathogenicity. However, it is undeniable that motility induced by the polar flagellum in *Vibrio* spp. contributes to the virulence of pathogenic *Vibrio* through adhesion or biofilm formation regardless of the environment. We are interested in understanding how the *Vibrio* flagellar structure and function are involved in the pathogenicity. We also want to know why and how this very complicated structure has evolved and been maintained in *Vibrio*.

### Conflict of interest statement

The authors declare that the research was conducted in the absence of any commercial or financial relationships that could be construed as a potential conflict of interest.
